# Predictors of infectious foci on FDG PET/CT in *Staphylococcus aureus* bacteremia

**DOI:** 10.1038/s41598-023-41336-6

**Published:** 2023-08-28

**Authors:** Nesrin Ghanem-Zoubi, Jawad Abu-Elhija, Olga Kagna, Mona Mustafa-Hellou, Majd Qasum, Daniel Haber, Mical Paul, Zohar Keidar

**Affiliations:** 1 Infectious Diseases Institute, Rambam Health Care Campus, Ha-Aliya 8 St, 3109601 Haifa, Israel; 2 The Ruth and Bruce Rappaport Faculty of Medicine, Technion, Israel Institute of Technology, Haifa, Israel; 3 Internal Medicine Department E, Rambam Health Care Campus, Haifa, Israel; 4 Department of Nuclear Medicine, Rambam Health Care Campus, Haifa, Israel; 5 Department of Cardiology, Rambam Health Care Campus, Haifa, Israel

**Keywords:** Diagnosis, Medical imaging

## Abstract

We looked for predicting factors for the detection of infectious foci on 18F-fluorodeoxyglucose-positron emission tomography in combination with computed tomography (FDG PET/CT) among patients with *Staphylococcus aureus* bacteremia (SAB) who participated in an interventional study that was conducted at Rambam Health Care Campus, between July 1, 2015 and February 1, 2019. The primary outcome was an infectious focus detected by FDG PET/CT. Independent predictors for detection of focal infection were identified using univariate followed by a logistic regression multivariate analysis. We included 149 patients with 151 separate episodes of SAB who underwent FDG-PET/CT. Focal infections were detected in 107 patients (70.8%). Independent predictors for focal infection detection were community acquisition of bacteremia with odds ratio (OR) 3.03 [95% confidence interval (CI) 1.04–8.77], *p*-0.042 and C reactive protein (CRP) with OR 1.09 [95% CI 1.04–1.14], *p* < 0.001. Primary bacteremia was inversely associated with focal infection detection with OR 0.27 [0.10–0.69], *p *= 0.007, as were the pre-scan blood glucose levels OR 0.9 [0.98–0.99], *p*-0.004. The latter stayed significant in the subgroup of patients with diabetes mellitus. To conclude, patients with community-acquired bacteremia or high CRP levels should be carefully investigated for focal infection. Patients who present with primary bacteremia seem to be at low risk for focal infection.

## Introduction

*Staphylococcus aureus* bacteremia (SAB) might arise from a focal source of infection or present as primary bacteremia. In both cases, distant seeding of the bacteremia to soft tissue, bone, joints, prosthetic devices and other locations is frequent^1^. In recent years, 18F-fluorodeoxyglucose-positron emission tomography computed tomography (FDG PET/CT) has been proposed as an imaging modality to diagnose deep foci of infection in SAB^2–5^. Recently, we demonstrated in an interventional study the added value of integrating FDG PET/CT in the diagnostic work up of patients with SAB^6^. In about 70% of patients who underwent PET/CT, at least one infectious focus was detected. Compared with matched controls, the use of PET/CT was independently associated with lower mortality (odd ratio (OR)) 0.39; 95% CI 0.18–0.84).

In the current study, we aimed to examine the predicting factors for infectious foci in SAB as detected by FDG PET/CT. These predictors may help in the stratification of patients' risk for focal infections who may benefit from routinely including FDG PET/CT in the diagnostic work.

## Methods

### Study design and location

Retrospective analysis of prospectively and retrospectively collected data of a patient cohort with SAB recruited prospectively to undergo FDG PET/CT as part of bacteremia investigation at Rambam Health Care Campus, a primary and tertiary 960-bed university-affiliated hospital, between July 1, 2015 and February 1, 2019^6^. The original study included adult patients (age > 18 years old) with SAB who underwent FDG PET/CT usually during the second week after infection onset. FDG PET/CT results were integrated in the treatment approach of SAB. The current study included all 149 patients with 151 separate episodes of SAB from the original cohort focusing on identifying the predictors of infectious foci detection by PET/CT.

### Dependent variable (outcome)

Detection of focal infection on FDG PET/CT. FDG PET/CT results were classified as positive for an infectious focus in the presence of pathological uptake suggestive of a focal infection such as osteomyelitis, joint infection (native and prosthetic), deep-seated abscesses, vascular infection or any other findings consistent with focal infection. The study was approved by Helsinki Committee of Rambam Health Care Campus in accordance with relevant guidelines and regulations. We included all patients who signed informed consent in the interventional study as well as patients who underwent FDG PET/CT according to physician decision for whom informed consent was waived^6^.

### Predictors

We collected data on patients' comorbidities, symptoms duration, duration of bacteremia and fever after starting appropriate treatment, clinical severity on bacteremia onset using the Pitt score, foreign bodies existence including central venous catheters. Laboratory parameters including the highest value of C reactive protein (CRP) from start of bacteremia to FDG PET/CT performance and the worst value of white blood cell count (highest in leukocytosis or lowest in leukopenia) on first 3 days following presentation were also collected. Place of acquisition was defined as nosocomial if bacteremia onset occurred > 48 h after admission, healthcare-associated if patients were on chronic hemodialysis or home intravenous treatment, or hospitalized in 90 days preceding index admission; otherwise, it was defined as community acquired. Primary bacteremia was defined in the absence of focal symptoms or signs of infection before FDG PET/CT. Polymicrobial bacteremia was defined if any other clinically-significant pathogen was isolated from blood cultures taken seven days pre/post start of SAB. Prolonged bacteremia was defined as positive blood culture after at least 72 h after start of appropriate treatment. High-risk SAB was defined if it was community acquired or patient had prolonged bacteremia or fever lasted above 72 h after start of appropriate treatment^7^.

### FDG-PET/CT acquisition, interpretation and analysis

PET/CT imaging (Discovery 690; GE Healthcare) was performed one hour after intravenous injection of 0.14 mCi/kg of F-18-FDG. Intravenous contrast media were also administered unless contraindicated. In suspected cardiac infection patients were instructed to keep a low carbohydrate, fat and protein-enriched diet for 12 h followed by a prolonged fasting of 12–14 h prior the PET/CT scan. Blood glucose levels were measured before administration of the radiotracer. Patients underwent eye-to-mid-thigh PET/CT acquisition, with head and lower limb scanning added when clinically indicated.

FDG PET/CT result was classified as positive for infectious focus in the presence of pathological increased FDG uptake suggestive of focal infection like osteomyelitis, joint infection (native and prosthetic), deep-seated abscesses, vascular infection, and others.

Final diagnosis was defined based on the whole clinical course integrating other imaging studies and additional microbiological results (isolation of *S. aureus* from samples taken from a suspected site of infection).

FDG PET/CT imaging was considered false negative in cases where a focal infection was documented by other modalities but no pathological finding was detected by PET/CT. False positive was defined if a positive focus was suggested by PET/CT focus but was excluded by other tests (i.e. a biopsy that yielded alternative diagnosis like malignancy), true positive if the FDG PET/CT focal infectious finding correlated well with the clinical course and true negative when no other tests yielded an infectious focus that was missed but we believed it existed at the time of FDG PET/CT performance.

### Data collection methods

Data were collected prospectively and complemented with retrospective data collected manually from patients' electronic medical records.

### Statistical analysis

Predictors of focal infection detection by FDG PET/CT were assessed by univariate and multivariate analysis. Categorical variables were compared using a Chi square or Fisher Exact test, ordinal variables by linear-on-linear Chi square and continuous variables using T-test or the Mann–Whitney U non-parametric test, as appropriate. Variables significant (*p* ≤ 0.05) on univariate analysis and not clinically or statistically correlated were entered into a back stepwise logistic regression model to assess the association with infectious foci detection by FDG PET/CT. Two models were performed, one with all significant variables and one omitting CRP measurements that were missing in about a third of patients. Sensitivity, specificity, positive and negative predictive values and likelihood ratios for PET/CT results compared to final diagnosis were calculated. Data analysis was performed using IBM SPSS Statistics version 28.0.

### Ethics approval

The study was approved by Helsinki Committee of Rambam Health Care Campus in accordance with relevant guidelines and regulations. We included all patients who signed informed consent in the interventional study as well as patients who underwent FDG PET/CT according to physician decision for whom informed consent was waived.

## Results

**Figure 1:**
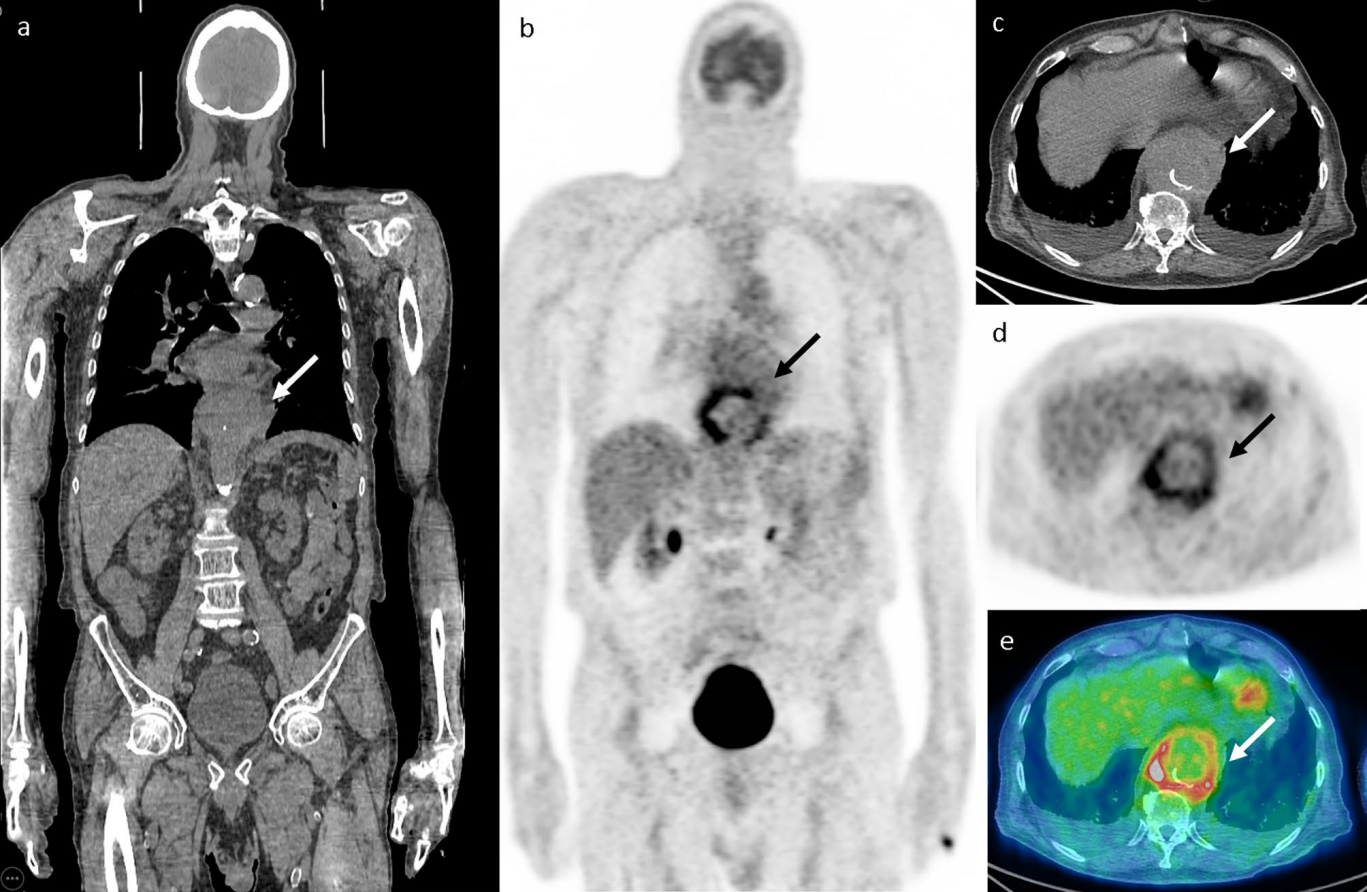
Infectious focus in an aortic pseudoaneurysm. A 73-year-old male presented with dyspnea and was diagnosed with *Staphylococcus aureus* bacteremia and empyema that was drained. FDG PET/CT images (**A**, **B** coronal slices, **C**, **D**, **E** axial slices at the level of the lower chest) show pathological FDG uptake in a thoracic aortic pseudoaneurysm (arrows). The patient underwent endovascular aortic stent insertion and was treated with intravenous antibiotics followed by chronic suppressive oral treatment. In a follow-up visit 6 years later, the patient has no evidence of disease.

149 patients with 151 separate episodes of SAB who underwent FDG-PET/CT were included in this study. FDG-PET/CT was performed at a median of 11^8,13^ days after the first positive blood culture. Positive findings suggestive of at least one infectious focus were observed in 107 episodes (70.8%). The most common foci were bone and joint infections observed in 58 (38.4%) of patients. Other foci involved skin/ soft tissue in 35 (23.1%), lungs in 32 (21.1%), vascular foci in 20 (13.2%), muscular foci in 16 (10.9%) and deep-seated abscesses and cardiac foci were observed in nine (5.9%) cases each^6^. Figure 1 shows an example of infectious focus in an aortic pseudoaneurysm.

### Predictors of pathological FDG uptake suspected for infection

Comparison between the 107 patients who had at least one infectious focus with 44 patients who had no detection of infectious foci according to PET/CT is presented in Table 1. The Charlson's comorbidity index was significantly higher in patients without detected infectious foci with a median of 8 [5, 10] compared to 5 [3, 7] in patients with detected infectious foci, *p* < 0.001. Significant differences in specific comorbidities were observed in the following: diabetes mellitus, peripheral vascular disease (PVD), history of cerebrovascular attack and chronic kidney disease, all were more prevalent among patients who had no detected infectious foci by FDG PET/CT. Community acquisition of bacteremia was more prevalent among patients with detected infectious foci where it was observed in 42 (39.3%) compared to 10 (22.7%) in patients without detected infectious foci, *p* = 0.05. Primary bacteremia was more common among patients without positive PET/CT findings occurring in 13 (29.5%) compared with 11 (10.2%) in the group with positive PET/CT focal infections, *p* = 0.003. CRP was available in 109 patients and was significantly higher in patients with infectious foci with a median of 23.74 [17.03, 31.18] mg/dl compared to 7.59 [3.93, 23.96], *p* < 0.001 in patients without infectious foci. No significant differences were observed in age, the presence of foreign bodies, immunosuppressive treatment, Pitt score and the presence of high risk SAB. In addition, the time duration between start of bacteremia (first positive blood culture), or start of appropriate treatment to PET/CT performance were similar in both groups. Blood glucose level before PET/CT was available in 149 cases. In the group with positive PET/CT for focal infection the mean of pre-scan blood glucose value was 109 ± 39 compared to 137 ± 70 in the PET negative group, *p*-0.002.

**Table 1:** Characteristics of patients with SAB who had findings suggesting complicated infection versus patients who had no findings suggesting complicated infection detected by FDG PET/CT.

Variable	Infectious focus detected by PET/CT N = 107	No infectious focus detected by PET/CT N = 44	*p* value
Baseline characteristics			
Age	58.85 ± 16.32	63.47 ± 15.12	0.108
Male	79 (73.8)	29 (65.9)	0.327
Community acquisition	42 (39.3)	10 (22.7)	0.052
Previous Hospitalization 90dys	51 (47.7)	23 (52.3)	0.607
Normal basic function status	90 (84.1)	33 (75.0)	0.190
Dementia	3 (2.8)	4 (9.1)	0.194
Comorbidities			
Charlson’s comorbidity score	5 [3, 7]	8 [5, 10]	0.001
Congestive heart failure	14 (13.1)	10 (22.7)	0.141
Cerebrovascular attack	7 (6.5)	7 (15.9)	0.043
Chronic renal failure	11 (10.3)	10 (22.7)	0.045
Diabetes mellitus	46 (43.0)	28 (63.6)	0.021
Chronic Pulmonary Disease	13 (12.1)	2 (4.5)	0.233
Peripheral Vascular Disease	7 (6.5)	10 (22.7)	0.004
Past myocardial infarction	26 (24.3)	17 (38.6)	0.076
Malignancy	7 (6.5)	7 (15.9)	0.071
Liver disease	3 (2.8)	1 (2.3)	1.000
Immunosuppressive treatment	23 (21.5)	15 (34.1)	0.105
Solid organ transplant	3 (2.8)	1 (2.3)	1.000
Intravenous drug user	5 (4.7)	0 (0.0)	0.322
Any prosthetic device	23 (21.5)	14 (31.8)	0.180
Any intra vascular catheter	22 (20.6)	10 (22.7)	0.767
Clinical presentation			
Pitt bacteremia score	2 [0,3]	2 [0,3]	0.682
Fever (temperature > = 38 °C)	69 (64.5)	33 (75.0)	0.210
Tachycardia (heart rate > 90/min)	84 (78.5)	28 (63.6)	0.058
Abnormal white blood cell count	60 (56.1)	22 (50.0)	0.496
C-reactive protein (mg/dl), (N = 109)	23.74 [17.03,31.18]	7.59 [3.93,23.96]	0.001
*Staphylococcus aureus*			0.763
Penicillin susceptible	27 (25.2)	11 (25.0)	
Methicillin susceptible	57 (53.3)	22 (50.0)	
Methicillin resistance	23 (21.5)	11 (25.0)	
Primary bacteremia	11 (10.2)	13 (29.5)	0.003
Polymicrobial bacteremia	8 (7.5)	4 (9.1)	0.746
Appropriate treatment within 24 h	56 (52.3)	24 (54.5)	0.805
Fever above 3 days after start appropriate treatment	13 (12.1)	1 (2.3)	0.067
Positive blood cultures > 3 days on appropriate treatment	32 (29.9)	12 (27.3)	0.746
Days from bacteremia onset to PET CT	11 [8, 14]	10 [8, 12]	0.36
Days from start of appropriate treatment to PET CT	10 [8, 13]	10 [8, 11]	0.15
Infective endocarditis	20 (18.7)	11 (25.0)	0.383
Echocardiography performed	104 (97.2)	42(95.5)	0.587
High risk SAB	63 (58.9)	21 (47.7)	0.21
Pre-scan blood glucose (mg/dl), n = 149	109 ± 39	137 ± 70	0.002

*SAB* *Staphylococcus aureus* bacteremia, *FDG PET/CT* F-fluorodeoxyglucose positron emission tomography.

A strong correlation was observed between diabetes mellitus and Charlson comorbidity index as well as the rest of comorbidities with significant differences in the univariate analysis. Therefore, diabetes mellitus was the variable chosen for evaluation in the multivariate analysis.

On multivariate logistic regression analysis including diabetes mellitus, community acquisition of bacteremia, primary bacteremia and glucose levels before PET, both primary bacteremia and pre-scan glucose levels were inversely associated with positive PET/CT focal infection with ORs of 0.27 [0.10–0.69], *p* = 0.007 and OR 0.9 [0.98–0.99] per 1 mg/dl increase in glucose, *p* = 0.004, respectively (Table 2). When including CRP in the model, significant variables were community acquisition OR 3.03 [1.04–8.77], *p* = 0.042; CRP with OR 1.09 [1.04–1.14] per 1 mg/dl, *p* < 0.001 and pre-scan blood glucose with OR 0.9 [0.98–0.99] per 1 mg/dl, *p*-0.004 (Table 3).

**Table 2:** Multivariate logistic regression analysis (without CRP).

Variable	OR [95% CI]	*p* value
Community acquisition	2.27 [0.97–5.31]	0.059
Diabetes mellitus	0.63 [0.27–1.51]	0.307
Primary bacteremia	0.27 [0.10–0.69]	0.007
Pre-scan blood glucose	0.98 [0.98–0.99]	0.003

**Table 3:** Multivariate logistic regression analysis (with CRP).

Variable	OR [95% CI]	*p* value
Community acquisition	3.02 [1.04–8.77]	0.042
Diabetes mellitus	0.53 [0.18–1.60]	0.266
Primary bacteremia	0.48 [0.12–1.85]	0.284
Pre-scan blood glucose	0.99 [0.98–1.00]	0.041
C-reactive protein	1.09 [1.04–1.14]	< 0.001

In both models, entering an interaction variable of pre-scan glucose levels by diabetes was statistically significant. Therefore, we sub grouped the cohort by diabetes: among patients without diabetes the pre-scan glucose level was not significantly associated with PET/CT findings (OR 1.01 [0.98–1.04], *p*-0.33), while among patients with diabetes mellitus, pre-scan blood glucose was significantly associated with positive infectious foci on PET/CT with an OR of 0.99 [0.98–0.99] per 1 mg/dl increase in glucose, *p* < 0.013.

Compared to final diagnosis (based on clinical course, other imaging results and additional microbiology data), PET/CT results accuracy were classified as true positive in 107, false positive in 0, true negative in 38 and false negative in eight patients. The overall sensitivity and specificity, positive predictive value and negative predictive value of PET/CT were 93%, 100%, 100% and 82%, respectively. Negative likelihood ratio was 0.07 [95% CI 0.04–0.14] (positive likelihood ratio could not be calculated with specificity of 100%). The eight patients with false negative results on FDG PET/CT had the following infectious foci: vascular catheter-related infection with a thrombus detected by echocardiography and positive cultures for *S. aureus* from catheter tip (three patients), soft tissue infection after below knee amputation (one patient), pulmonary infection with a positive concomitant culture from bronchoscopy and lung infiltrate (one patient), obstructed pyelonephritis and a drained pus from kidney that grew same bacterium as in the blood culture (one patient), mediastinitis with positive tissue cultures (one patient) and right sided IE without uptake in the heart but FDG uptake in suspected septic emboli in lungs (one patient). Six of these eight patients with false negative PET/CT results had diabetes mellitus.

## Discussion

In present study we found that community acquisition of bacteremia and elevated CRP were independently associated with the detection of infectious foci in SAB, while primary bacteremia and increased pre-scan blood glucose level were significantly associated with lack of findings on FDG PET/CT.

Our finding on community-acquired SAB is consistent with results from previous studies that reported community-acquired SAB to be associated with complicated infections^7,8^. The longer time until diagnosis in community compared to nosocomial infection and the delay in the start of treatment might allow for hematogenous seeding. As in our study, several studies have reported the association between elevated CRP levels and complicated SAB, deep-seated infections and even higher mortality^9–12^. Higher CRP values may indicate a more aggressive and extensive infection. A pre-test diagnosis of primary bacteremia was mostly confirmed on PET/CT in our study that ruled out a focus of infection. Further studies with large cohorts are needed in order to characterize this low-risk group of patients.

Increased pre-scan blood glucose levels among patients with diabetes mellitus were inversely associated with detection of infectious foci in our study. This finding might explain the high proportion of patients with diabetes (6 out of 8, 75%) in the group of patients with false negative PET/CT results. The lower diagnostic accuracy of FDG PET/CT in patients with diabetes, acute or chronic hyperglycemia had been discussed in several previous studies^13–19^. Blood glucose levels before FDG PET/CT rather than diabetes mellitus per se seems to be the factor affecting FDG uptake^13,16,17^. One proposed explanation for the lower sensitivity, is the disruption for FDG uptake by the cells due to down regulation of glucose uptake receptors. The differences in bio distribution between different organs and the specific organ used as reference for standardized uptake value (SUV) measurement is in the center of the ongoing argument in oncology patients^16–18,20^. In one study, the correlation between blood glucose level and sensitivity of FDG PET/CT was observed in oncological patients but not in infectious/inflammatory patients^13^. Controlling acute changes in blood glucose levels in severe acute infection may be highly challenging, while the use of insulin immediately prior to PET/CT may also adversely affect the detection rate of pathological processes^19^. The role of glycaemia control before FDG PET/CT in the investigation of infections should be further evaluated. Nevertheless, the current study shows overall high specificity and sensitivity of FDG PET/CT in identifying SAB foci.

Our study has several limitations. Data collection was retrospective, from a single center. PET/CT was intentionally performed 7–14 days after the SAB onset; the PET/CT results might have been affected by the antibiotic treatment, although no difference in duration of antibiotic. treatment was observed in our study. In addition, the patients' diagnosis might have been affected by other tests performed before the PET/CT. Lab tests, mainly CRP, were taken according to physicians' discretion in a non-standardized protocol, thus data were missing. The advantage of the current cohort is the prospective performance of PET-CT to all consecutive consenting patients with SAB, allowing us to analyze predictors for positive findings without pre-test patient selection.

In conclusion, our study confirms the complicated nature of community-acquired SAB and the value of CRP in predicting a complicated course. Nevertheless, patients who present with primary bacteremia seems to be at low risk for focal infection.

## Data Availability

The datasets generated during and/or analysed during the current study can be achieved by contacting the corresponding author.
